# Structural insights into *Cydia pomonella* pheromone binding protein 2 mediated prediction of potentially active semiochemicals

**DOI:** 10.1038/srep22336

**Published:** 2016-03-01

**Authors:** Zhen Tian, Jiyuan Liu, Yalin Zhang

**Affiliations:** 1 Key Laboratory of Plant Protection Resources & Pest Management of the Ministry of Education, College of Plant Protection, Northwest A&F University, Yangling 712100, Shaanxi, China

## Abstract

Given the advantages of behavioral disruption application in pest control and the damage of *Cydia pomonella*, due progresses have not been made in searching active semiochemicals for codling moth. In this research, 31 candidate semiochemicals were ranked for their binding potential to *Cydia pomonella* pheromone binding protein 2 (CpomPBP2) by simulated docking, and this sorted result was confirmed by competitive binding assay. This high predicting accuracy of virtual screening led to the construction of a rapid and viable method for semiochemicals searching. By reference to binding mode analyses, hydrogen bond and hydrophobic interaction were suggested to be two key factors in determining ligand affinity, so is the length of molecule chain. So it is concluded that semiochemicals of appropriate chain length with hydroxyl group or carbonyl group at one head tended to be favored by CpomPBP2. Residues involved in binding with each ligand were pointed out as well, which were verified by computational alanine scanning mutagenesis. Progress made in the present study helps establish an efficient method for predicting potentially active compounds and prepares for the application of high-throughput virtual screening in searching semiochemicals by taking insights into binding mode analyses.

For most insects, such fundamental behaviors as mating, predation, and oviposition as well as avoiding threats are controlled by small molecule signals. Former research has revealed that olfaction system played a significant role in detecting chemical signals^1^,^2^. In the olfaction system, to reach the membrane containing pheromone receptors, hydrophobic pheromones have to cross the aqueous sensillum lymph surrounding the dendrites of neuronal cells^3^. The crossing of this aqueous barrier is thought to be assisted by pheromone binding proteins (PBPs)^4^,^5^,^6^.

Insect PBPs, as a class of odorant binding proteins, are small soluble proteins rich in the lymph of pheromone-sensitive sensilla, in the Sensilla Trichodea of Lepidoptera, the concentration can reach10–20 mM^4^,^7^. Since their first identification by binding to radio-labelled pheromone in the giant silk moth *Antheraea polyphemus*, PBPs have been identified in various lepidopteran species^4^,^8^,^9^,^10^.

Olfaction systems are of high sensitivity and specificity^11^. It could be estimated, according to wind tunnel experiments, that about 200 molecules were sufficient enough to elicit a behavioral response; EAG experiments also showed that a single molecule of pheromone can be transducted to an electrical signal^12^. Recent experimental evidence, both from behavioral and molecular biology, may support a more specific role of PBPs in detecting and recognizing semiochemicals^13^,^14^,^15^. Insects can correctly recognize pheromones from a mass of physiologically irrelevant chemical compounds, but even a minimal modification can result in the inactivity of pheromones^16^,^17^. However, *in vitro* binding experiments of PBPs indicated that some lepidopteran PBPs like BmorPBP from *Bombyx mori*, ApolPBP from *Antheraea polyphemus* and LdisPBP from *Lymantria dispar* exhibited high affinity to more than one ligand, pheromones included^18^,^19^. To some degree, this promiscuity could be explained, since sacrificing specificity for sensitivity is reasonable in the early events of olfaction.

The binding and release mechanism of PBPs is always an important issue. Lepidopteran PBPs were suggested to have two conformations in a pH/ligand-dependent manner. The A-form (closed or free form) characterized by the seventh α-helix formed by the C-terminus, was observed in the absence of pheromone and/or at low pH, whereas the B-form (open or bound form) characterized by the unstructured C-terminal tail, was detected in the presence of ligand at high pH^20^,^21^,^22^. These results in combination with the lower pH generated by negatively charged membrane surfaces led to the promotion of a pH-induced ligand-releasing mechanism, following which the BmorPBP released the bound bombykol when encountering the lower pH around the receptors^17^. A similar phenomenon was also observed in ApolPBP^23^. Additionally, Katre *et al.* pointed out that the C-terminus in ApolPBP1, forming the seventh α-helix, played an important role in ligand binding and/or locking the ligand in the binding pocket as well^21^.

Even though several protein classes, including odorant receptors (ORs), odorant binding proteins (OBPs), sensory neuron membrane proteins (SNMPs) and odorant degrading enzymes (ODEs), have been reported to be involved in insect odorant perception^24^,^25^,^26^,^27^, the best studied OBPs were thought as appealing targets for structure-based prediction of physiologically active semiochemicals. Since the promotion of reverse chemical ecology, several attempts have been made either to screen odorants or to study ligand-binding mechanism on the basis of modeling OBP structure^28^,^29^,^30^.

The codling moth *Cydia pomonella*, is a quarantine insect causing great harm to a variety of pome fruits every year in China. Today, pesticide applications are being called for diminishing. Therefore to control codling moth, the use of odorants for behavioral disruption is the most often alternative to pesticides^31^. However, very little is known about the chemoreception system of the codling moth. These days, studies on the codling moth PBPs mainly focused on searching new PBPs^32^, no reports on functional analysis and structure of CpomPBPs have been found.

In this article, the *Cydia pomonella* pheromone binding protein 2 (CpomPBP2) was cloned and expressed to study its biochemical characterizations. More importantly, in order to construct a rapid way for searching semiochemicals, virtual screening and an *in vitro* binding assay were used in combination with reference to the notion of “reverse chemical ecology”^28^,^30^,^31^. The 3D structure of CpomPBP2 was initially modeled and used for molecular docking to screen semiochemicals. Competitive binding assay was applied to test the prediction accuracy of our computational method. Moreover, on the account of preparing for the application of high-throughput virtual screening, we also tried to find out key factors affecting affinity by analyzing the binding modes of the CpomPBP2-ligand complex.

## Results and Discussion

### CpomPBP2 protein characteristics and tissue distribution

To analyze the phylogenetic relationship of CpomPBP2 with other lepidopteran insects, we constructed a phylogenetic tree comprising 28 lepidopteran PBPs from different families (see Supplementary Fig. S1). CpomPBP2 was phylogenetically closest to GmolPBP2 from *Grapholitha molesta* and LglyPBP2 from *Leguminivora glycinivorella*, both of which belong to the Olethreutidae. This result was in line with the classification of *Cydia pomonella* (Tortricidae: Olethreutinae) in Lepidoptera.

In this research, to make CpomPBP2 and TPBP2 express in the supernatant, the expression systems PET28a(+)/CpomPBP2 and PET28a(+)/TPBP2 were transformed into the Rosetta-gami 2 (DE3) competent cells. Compared with strains like BL21 (DE3), Rosetta gami 2 promotes the expression of soluble protein. The expected bands were detected at about 20 KD and 18 KD (see Supplementary Fig. S2C), as the molecular weights of CpomPBP2 and TPBP2 were 16.5 KD and 14.5 KD, and the PET28a(+) tag was about 4 KD. By using a Ni^2+^-NTA agarose gel column (Transgen, China), the target recombinant protein was purified and analyzed on 15% SDS-PAGE gel. The presence of the additional N-terminal sequence containing the His tag slightly increased molecular weight and modified the isoelectric point of the recombinant CpomPBP2, but did not affect the functional property of ligand binding^33^,^34^.

Reverse-transcription PCR (RT-PCR) was applied to check the distribution pattern of CpomPBP2 expression. The objective product was specifically detected in male and female antennae (see Supplementary Fig. S2A), and this result has been verified by western blot analysis of native CpomPBP2 (see Supplementary Fig. S2B). This is also in accordance with former reports in other lepidopteran insects^9^,^10^,^35^,^36^.

### 3D structure modeling

**Figure 1:**
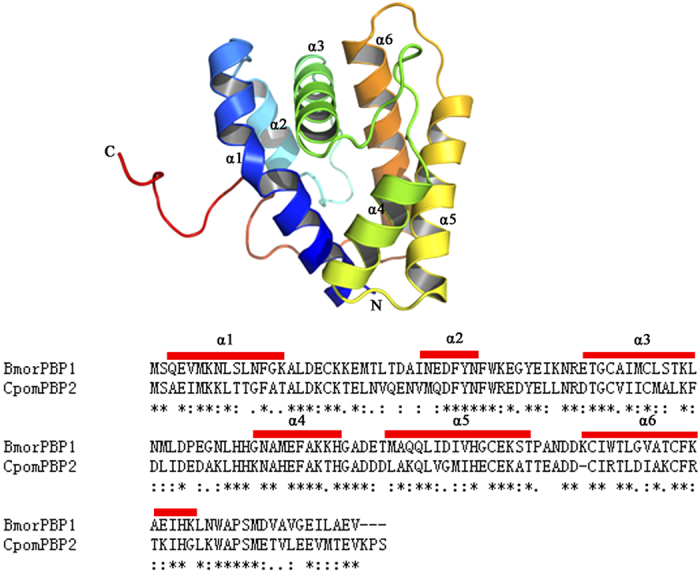
Structure of CpomPBP2. (**A**) 3D structure of CpomPBP2. N is the N-terminus, C is the C-terminus, and helices α1-α6 as labeled, of which α1, α4, α5 and α6 converge to form the binding pocket. (**B**) Strucutre-based sequence alignments of *Cydia pomonella* CpomPBP2 and *Bombyx mori* BmorPBP obtained with Clustal W and refined using the CpomPBP2 structure. The identical residues are highlighted with star below the letters. The six predicted α-helices are marked on the top of sequences using red boxes.

As shown in Fig. 1, CpomPBP2 shared 50% sequence identity with the selected template (PDB ID: 1DQE). After 100 CpomPBP2 models calculation by MODELLER, the CpomPBP2 model with the lowest DOPE assessment score was selected. 3D quality of the best model was assessed by the Ramachandran Plot (see Supplementary Fig. S3). The result revealed that 99.3% (141/142) of all residues were in favored (98%) regions, 100.0% (142/142) of all residues were in allowed (>99.8%) regions, and there were no outliers. The best model of CpomPBP2 was also verified by Profile 3D^37^. According to Supplementary Fig. S4, 88.89% of the residues had an average 3D-1D score >= 0.2, and at least 80% of the amino acids have scored >= 0.2 in the 3D/1D profile. All of these parameters suggest that the 3D structure of CpomPBP2 is rational and can be used for further virtual screening.

The 3D structure of CpomPBP2, as shown in Fig. 1, was formed by a roughly conical arrangement of six α helices connected by loops. Four antiparallel helices (α1, α4, α5 and α6) converged to form a large flask-shaped binding pocket with the narrow end being open and the opposite end being capped by the α3 helix.

Furthermore, conformation of the best predicted model of CpomPBP2 was checked by intrinsic fluorescence analyses^18^,^38^. The corresponding emission spectra of CpomPBP2 peaked at 337 nm when excited at 250 nm (see Supplementary Fig. S5). Former researches showed that the fluorescence maximum for free tryptophan in water and Trp residues in unstructured peptides was reached at 350 nm and shifted to shorter wavelengths in more hydrophobic environment^18^. In the present study, the detected peak at 337 nm was consistent with a mixture of hydrophobic and solvent exposed environments of the two Trp residues. This was in accordance with the predicted results when modeling the CpomPBP2 sequence on the crystal structure of BmorPBP-bombykol complex with one (Trp37) in a hydrophobic environment and the other (Trp127) being more solvent exposed.

We also detected that fluorescence intensity of CpomPBP2 increased along with pH amplification (see Supplementary Fig. S5). This pH-dependent intensity change was similar to the counterparts of BmorPBP and ApolPBP^18^,^33^, indicating that CpomPBP2 may possess similar conformational changes to these two proteins which have been suggested to have two major conformations in a pH/ligand-dependent manner by Circular Dichroism, tryptophan fluorescence as well as NMR and Crystal structure^5^,^17^,^18^,^20^,^22^,^33^.

### Virtual screening and prediction of active semiochemicals

31 compounds including bombykol (see Supplementary Table S1) were subjected to molecular docking simulations. After docking calculations by GOLD 5.3, the accurate binding mode of CpomPBP2-bombykol was obtained, and the superimposition of the conformations between CpomPBP2-bombykol and BmorPBP-bombykol complexes is shown in Supplementary Fig. S6. The superimposition RMSD (root-mean-square deviation) with the value of 0.113 Å between the conformations of the bombykol in BmorPBP and CpomPBP2 indicated that the docking works of the whole compounds were accurate in the binding pocket of CpomPBP2. Recent studies demonstrated that ChemPLP was superior to other scoring functions in GOLD program for pose prediction, only based on the accurate binding mode can the accurate affinity sequence be obtained^39^,^40^. So the score function of ChemPLP fitness and the binding free energy changes (ΔG) derived from Chemscore were considered in measuring the activity of compounds (see Supplementary Table S1).

Given the ChemPLP fitness and ΔG in Table S1 and commercial availability, 13 ligands (marked with * in Supplementary Table S1) were finally chosen to carry out the competitive binding assay under pH7.0 to validate whether the predicted compounds were active or not.

**Figure 2:**
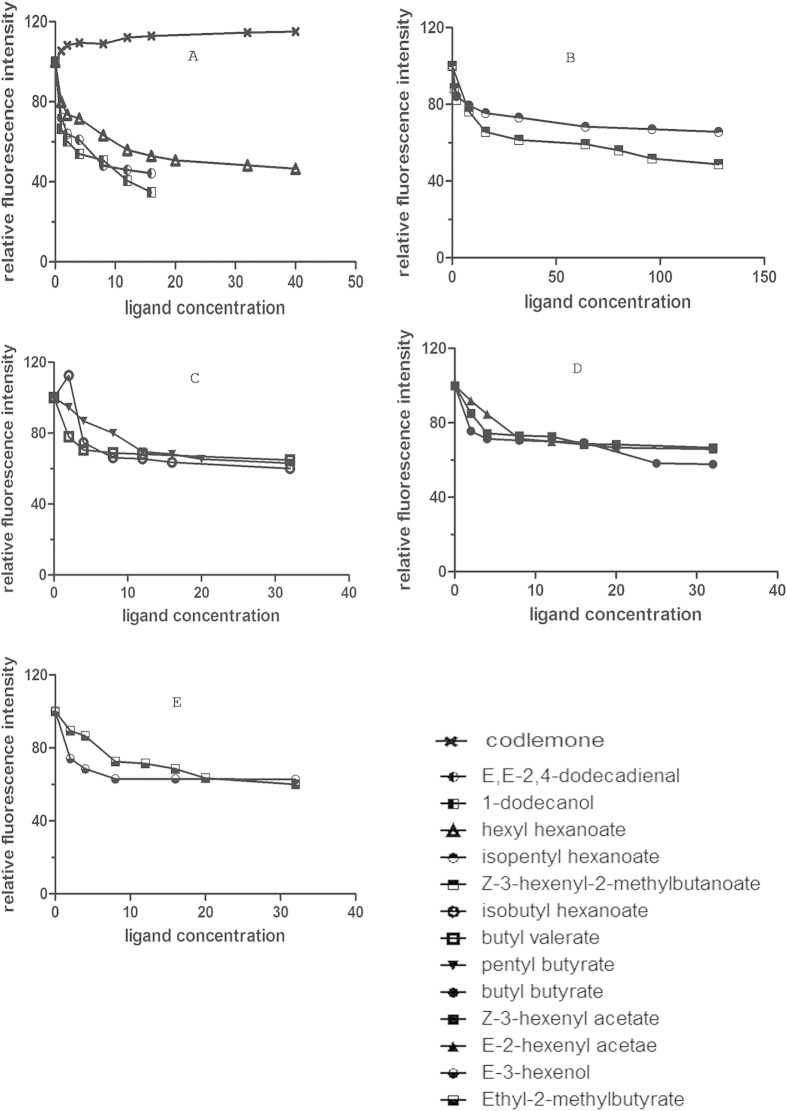
Competitive binding curves of some ligands to CpomPBP2. Solutions (50 mM Tris-HCl pH 6.8) containing CpomPBP2 and 1-NPN both at 2 μM, were titrated with increasing amounts of competing ligands. (**A**) Ligands applied consisted of 12C-Skeleton odorants, (**B**) 10C-Skeleton odorants, (**C**) 9C-Skeleton odorants, (**D**) 8C-Skeleton odorants and (**E**) 6C-Skeleton odorants.

The binding curve of 1-NPN and the Scatchard plot (see Supplementary Fig. S7A) indicated a dissociation constant (*K*_*D*_) of 7.846 μM. According to the results of competitive binding assay, four compounds were found to be able to quench the fluorescence intensity of 1-NPN/CpomPBP2 complex to 50% (see Fig. 2). The dissociation constants of these four compounds, 1-Dodecanol, E,E-2,4-Dodecadienal, Hexyl Hexanoate, and Z-3-Hexenyl-2-Methylbutanoate, were 4.14 ± 0.29 μM, 6.99 ± 0.72 μM, 16.20 ± 0.84 μM, and 97.03 ± 2.26 μM respectively (see Table 1). As it is known that smaller *K*_*D*_ always indicates higher affinity, this obtained sequence was in qualitative agreement with what we predicted through molecular docking (see Table 1). What should be noted is that, except for Z-3-hexenyl-2-methylbutanoate, the selected odorants with smaller *K*_*D*_ are characterized by a 12C-skeleton. This difference in the length of the C-skeleton may contribute to the low binding affinity of Z-3-hexenyl-2-methylbutanoate.

**Figure 3:**
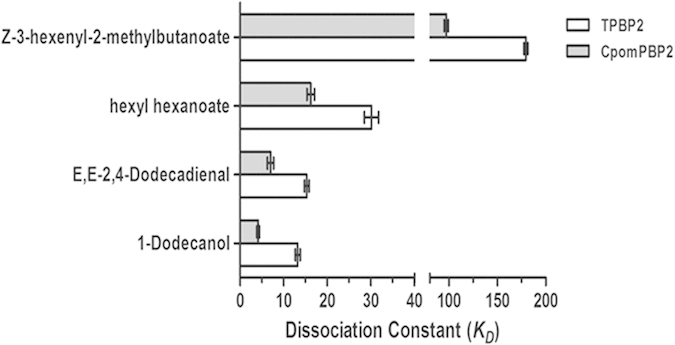
Effects of C-terminus on the binding of ligands to CpomPBP2. Deletion of the C-terminus of CpomPBP2 largely decreased the affinity between ligands and protein.

Apart from the length of the C-skeleton, pH is an external factor in affecting ligand affinity. As expected, when pH became 5.0, no tested ligands could be detected binding to CpomPBP2 (data not show). A conformational switch of the C-terminus at one end of the binding pocket was reported to play an important role in this process^20^,^21^,^22^. Binding assays suggested that the binding affinity at pH7.0 was evidently declined when changing CpomPBP2 into TPBP2 (see Fig. 3). Unlike CpomPBP2, binding of these four ligands to TPBP2 was not affected by pH variation (data not shown). TPBP2 was far from alone with this respect^21^,^41^. Nevertheless, the truncated form of BmorPBP and ApolPBP behaved otherwise with the binding affinity not being affected by truncation of the C-terminus at physiological pH^16^,^42^. These contradictory views suggest different uploading mechanisms of the pheromone^21^,^43^. More importantly, our results verified the essential role of the C-terminus in ligand binding/uploading as well.

The dissociation constants above exhibited poor discrimination of CpomPBP2 to molecules, CpomPBP2 is never an individual example in this aspect^19^,^44^. This promiscuity could be regarded as a form of olfaction sensitivity. After all, In the early events of olfaction, sacrificing certain specificity for sensitivity is reasonable and necessary^17^. Our results also provide evidences for the concept of a two-step filter in which specific detection of pheromone is mediated by both PBPs and pheromone receptors^44^,^45^,^46^. Some reports demonstrated that the specific conformation of PBP-ligand complex also has a role in pheromone discrimination^18^,^44^,^47^. In the case of *Drosophila* LUSH, only the specific pheromone can trigger the appropriate conformational change, allowing it to be recognized by the specific receptor, even though LUSH was able to bind different nonspecific ligands with similar affinity to that of the specific one^14^.

### Binding mode and binding free energy analyses

**Figure 4:**
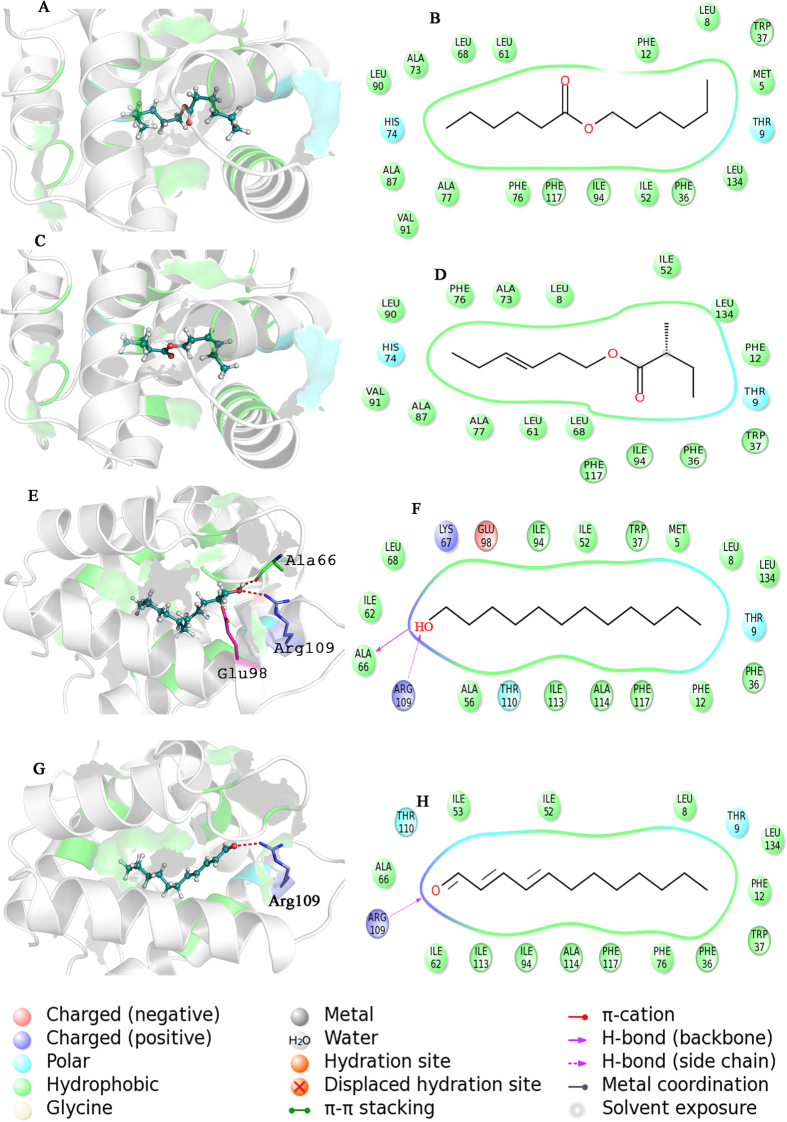
The carton representation of CpomPBP2 3D model around the binding site of all odorants. The binding model and interaction diagram of CpomPBP2 with Hexyl hexanoate (**A,B**), with Z-3-hexenyl-2-methylbutanoate (**C,D**), with 1-Dodecanol (**E,F**) and with E,E-2,4-dodecadienal (**G,H**). The four odorants are presented as stick and sphere model. *Cyan* C, *White* H, *Red* O. the red dotted lines show hydrogen bonds among the atoms from amino acid residues and odorants.

In order to further investigate the characteristics of binding at the structural level, four active compounds (Table 1) were selected to perform an analysis for binding mode and binding free energy. As shown in Fig. 4A,C, Hexyl Hexanoate and Z-3-Hexenyl-2-Methylbutanoate possessed similar binding modes, and were bound in the large hydrophobic pocket whose component residues including Leu8, Phe12, Trp37, Ile52, Leu61, Leu68, Phe76, Leu90, Val91, Ile94, Phe117 and Leu134 were involved in binding to these two compounds (see Fig. 4B,D). Both of these compounds formed polar interactions with residues Thr9 and His74 as well. By comparison, we found that Hexyl Hexanoate was provided with a larger hydrophobic contact area than Z-3-Hexenyl-2-Methylbutanoate, especially at the site of Leu90 and Phe117 (see Fig. 4A,B). This phenomenon corresponded to the individual energy terms derived from binding free energy analysis shown in Table 1, the compound Hexyl Hexanoate possessed a more favorable hydrophobic interaction energy contribution [S(lipo)] than Z-3-Hexenyl-2-Methylbutanoate, leading to more binding free energy change and higher affinity (lower *K*_*D*_) of CpomPBP2-Hexyl Hexanoate complex.

Different from the two compounds above, 1-Dodecanol and E,E-2,4-Dodecadienal formed hydrogen bonds and charged interactions within the binding pocket of CpomPBP2 as shown in Fig. 4E,G, even though similar polar interactions with Thr9 and Thr110 were also found in CpomPBP2-Dodecanol and CpomPBP2-Dodecadienal complexes. For E,E-2,4-Dodecadienal, a hydrogen bond was established between the oxygen atom derived from the carbonyl group of the compound and the NH atom from the side chain of Arg109 with an O-N distance of 3.0 Å (see Fig. 4G,H). Meanwhile, charged interaction between Arg109 and E,E-2,4-Dodecadienal was also detected (see Fig. 4G,H). In the CpomPBP2-Dodecanol complex, the hydroxyl group of 1-Dodecanol formed a hydrogen bond with the side chain of Arg109 with the distance 2.9 Å (see Fig. 4E,F). What quite different from the complex formed by CpomPBP2 and E,E-2,4-Dodecadienal was that a second hydrogen bond was established between the hydroxyl group and the main chain of Ala66 whose distance was 1.6 Å (see Fig. 4E,F). Moreover, the hydroxyl group of 1-Dodecanol formed positive and negative charged interactions with Lys67 and Glu98. These were in perfect agreement with the binding free energy changes and individual energy terms for 1-Dodecanol and E,E-2,4-Dodecadienal listed in Table 1. The CpomPBP2-Dodecanol complex exhibited over 1 KJ/mol more binding free energy change than CpomPBP2-Dodecadienal complex. The individual energy contributions in Table 1 showed that, corresponding to the relatively stronger hydrogen bond interactions in 1-Dodecanol, the hydrogen bond energy item contribution [S(hbond)] of 1-Dodecanol (1.27) was much greater than that of E,E-2,4-Dodecadienal (0.95). Interestingly, by comparing the chemical scaffold between 1-Dodecanol and E,E-2,4-Dodecadinal, we found that the saturated aliphatic chain group derived from 1-Dodecanol (207.74) was provided with a larger hydrophobic energy item contribution [S(lipo)] than the olefin group in E,E-2,4-Dodecadienal (192.24).

All of these findings suggested that both hydrogen bond energy item and hydrophobic interaction energy item had something to do with the binding affinity of these four compounds to the active pocket of CpomPBP2. As shown in Table 1 and Supplementary Table S1, It seemed that compound owning long aliphatic chain group was provided with remarkable hydrophobic interaction for the binding. It was assumed that shorter chain may result in smaller surface area of interaction and poorer binding ability^29^. So compounds of saturated 12C-skeleton tended to exhibit higher affinity to CpomPBP2 than shorter/unsaturated-chain compounds.

### Computational alanine scanning mutagenesis

To verify the key residues identified in the simulation docking procedure, the CpomPBP2/1-Dodecanol complex with the highest affinity was subjected to the computational alanine scanning (CAS) mutagenesis^48^. According to the per-residue energy decomposition, four key residues Phe12, Glu98, Arg109 and Ile113 (Figure S8), whose side chains contributed more than 1 kcal/mol to the energy change, were chosen for mutation to alanine based on the 10ns molecular dynamics trajectories.

The CAS results of the key residues were shown in table S2. The mutation of Arg109 to Ala caused the largest change in binding free energy (ΔΔG_bind_ = 6.25 kcal/mol). The binding free energy also dropped dramatically when the residue Glu98 was mutated to alanine (ΔΔG_bind_ = 5.34 kcal/mol). The ΔΔG_bind_ lower than 4 kcal/mol was observed on mutating the residues Phe12 (ΔΔG_bind_ = 3.17 kcal/mol) and Ile113 (ΔΔG_bind_ = 2.66 kcal/mol).

By reference to the definition of hot-spots and warm-spots^48^,^49^, two residues (Arg109 and Glu98) met requirements of hot-spots, whereas the other two fitted with warm-spots. The CAS method achieved an overall success rate of 80% and an 82% success rate in residues whose alanine mutation caused an increase in the binding free energy > 2.0 kcal/mol (warm- and hot-spots)^48^. Our CAS results could be regarded as verification to the results of simulation docking.

## Conclusions

By taking virtual screening and *in vitro* binding assay together, we provided an efficient and viable method for screening active semiochemicals. Of the 13 ligands applied, results of both methods (*in vitro* binding assay and molecular docking) indicated that 1-Dodecanol was most favored by the binding pocket of CpomPBP2, with E,E-2,4-Dodecadienal, Hexyl Hexanoate and Z-3-Hexenyl-2-Methylbutanoate less favored. The agreement between the results of competitive binding assay and virtual screening suggested the high prediction accuracy of the computational method we applied.

Binding mode analyses revealed much information on the interactions between ligands and CpomPBP2. Key residues involved in interacting with these four ligands were pointed out. The computational alanine scanning mutagenesis also confirmed the confidence of key residues identified in binding mode analyses. For CpomPBP2-ligand complex, we found that hydroxyl group with higher S(hbond) contributed more to binding free energy change than carbonyl group, saturated aliphatic chain of 12-C skeleton owned larger hydrophobic interaction than unsaturated or shorter chain, this characteristic made ligands of long saturated chain tend to exhibit higher affinity to CpomPBP2 than shorter/unsaturated ones. Considering individual energy terms and the sequence of affinity in Table 1 in combination, it could be estimated that hydrogen bond and hydrophobic interaction were key factors in determining the binding affinity of ligands to CpomPBP2. This work is of guiding importance to potentially active semiochemicals screening and makes high-throughput virtual screening become available.

## Methods

### Sample collection and RNA extraction

Codling moths *C. pomonella* were reared at 27 °C, in 16h light : 8h dark on an artificial diet in the laboratory. The antennae of 3d-old male and female adults were excised at the base and immediately transferred into tubes immersed in liquid nitrogen. The preparation of all other samples including the egg, the 1st to 5th instar larvae, pupae, head (without antennae), thorax, abdomen, leg and wing were carried out in the same way. Prepared samples were stored at −80 °C. The total RNA of each sample was isolated according to the manual of RNAiso Plus (TaKaRa, Japan). First-strand cDNA was synthesized by reference to the handbook of RevertAid First Strand cDNA Synthesis Kit (Thermo, USA) and employed as templates for latter PCR amplification.

### Phylogenetic analysis

To conduct the phylogenetic analysis of CpomPBP2 (GenBank: JQ776635), 28 lepidopteran PBPs available in NCBI were downloaded and aligned using ClustalX, MbraPBP1 (*Mamestra brassicae*, AAC05702), MbraPBP2 (*M. brassicae*, AAC05701), BmorPBP (Bombyx mori, NM_001044029.1), SnonPBP2 (*Sesamia nonagrioides*, AAS49922), AipsPBP2 (*Agrotis ipsilon*, AAX85459), HvirPBP2 (*Heliothis virescens*, CAL48346), HassPBP3 (*H. assulta*, ABB91374), MsexPBP2 (*Manduca sexta*, AAF16710), AtraPBP2 (*Amyetois transitella*, ACX47892), LglyPBP2 (*Leguminivora glycinivorella*, AEO91540), GmolPBP2 (*Grapholitha molesta*, AHZ89398), AhetPBP2 (*Atrijuglans hetaohei*, AKA27976), PxylPBP2 (*Plutella xyllostella*, AGH13203), DplePBP3 (*Danaus plexippus*, EHJ71308), SinfPBP2 (*Sesamia inferens*, AEX58642), SinfPBP3 (*Sesamia. inferens*, KF960746), SexigPBP2 (*Spodoptera exigua*, AAS55551), SexigPBP3 *(Spodoptera exigua*, ACY78413), CsupPBP2 (*Chilo suppressalis*, ACJ07123), ApolPBP2 (*Antheraea polyphemus*, AJ277266), AperPBP2 (*Antheraea pernyi*, X96860), SlituPBP2 (*Spodoptera litura*, ABK41048), XcniPBP (*Xestia cnigrum*, AGS41497), HzeaPBP (*Helicoverpa zea*, AF090191), HviriPBP (*Heliothis viriplaca*, AFI25170) and HarmPBP1 (*Helicoverpa armigera*, AEB54585). The phylogenetic tree was constructed by the MEGA4 program using the neighbor-joining method and bootstrapping sampled 1000 times.

### Distribution pattern of CpomPBP2

Tissue distribution of CpomPBP2 was assessed by RT-PCR with cDNA templates from different tissues of female and male moths, cDNA templates from egg, the 1st to the 5th instar larvae and pupae were also subjected to RT-PCR analysis. Specific primers, PBP2F and PBP2R (see Table 2), were employed. For testing the integrity of the cDNA templates, a control primer pair (see Table 2) from the coding region of the *Cydia pomonella β-actin* gene (GenBank: KC832921) was used.

### CpomPBP2 expression and purification

CpomPBP2 gene containing endonuclease restriction sites was cloned (EP1F and EP1R as primers) and incorporated into PET-28a(+), and the generated constructs were transformed into *E. coli.* Rosetta gami 2 (DE3) strains. After 18hrs’ induction with 0.6 mM IPTG under the condition of 16 °C, 160rpm, purification of the recombinant CpomPBP2 was initiated by centrifugation harvesting of 1L CpomPBP2-expressing DE3 cultures. Periplasmic fractions were prepared according to the osmotic shock procedure in the manual (Novagen). These obtained periplasmic fractions were centrifuged at 12000 g for 30 min, the supernatants were loaded onto Ni^2+^-NTA sepharose gel columns (Transgen, China) and eluted following the manufacturer’s instructions. Purified CpomPBP2 was analyzed by SDS-PAGE and dialyzed against 10 mM PBS, pH7.4.

C-terminal tail truncated form of CpomPBP2 (TPBP2) was also expressed by removing the last 17 amino acids PSMETVLEEVMTEVKPS. A pair of primers, TP2F and TP2R (see Table 2), were applied to obtain the expression-ready gene. The following steps were the same as mentioned above.

### Western blot analysis

Polyclonal antibodies against CpomPBP2 were produced by injecting rabbits. To test the expression pattern of CpomPBP2, Total protein extracted from tissues including antenna, head, thorax, abdomen, leg and wing of 3d-old moths by homogenizing in 20 mM Tris-HCl (pH7.4). Extracted proteins of the same quantity were separated by 15% SDS-PAGE, and the proteins were transferred to NC membranes by a semi-dry transfer cell (Biorad, USA). After being blocked for 3h with 5% skimmed milk in TBST (0.05% Tween-20 in TBS), the NC membrane was incubated for 2h with the polycolonal antibody against CpomPBP2 at a dilution of 1:8000, several washes with TBST followed. Thereafter, the second antibody, goat anti-rabbit IgG conjugated with HRP (Jackson, USA), was applied at a dilution of 1:10000, the immunoreaction bands were detected using an ECM kit (Boster, China).

### Structure modeling

The program Modeller 9.10 was applied to construct the 3D molecular model of CpomPBP2^50^. After searching for the PDB95 database with the amino acid sequence of CpomPBP2 being a probe, the crystal structure of the BmorPBP-bombykol complex from *Bombyx mori* (PDB ID: 1DQE, Chain A, resolution 1.8 Å) was selected as a template on the basis of the crystallographic R-factor (21.8%), the sequential identity (50%) and the pH state (pH7.0). The 3D model of CpomPBP2 was generated and refined using the automodel and loopmodel modules in the Modeller program respectively. The obtained model was also subjected to the GA341 and discrete optimized energy (DOPE) scores to measure the relative stability of CpomPBP2 conformation. The credible structure of CpomPBP2 with the lowest DOPE energy was selected and its quality was assessed by using MolProbity to identify the rationality of the stereochemistry for the structure^51^.

### Intrinsic fluorescence detection

Intrinsic fluorescence analysis was taken to verify the reliability of the simulated 3D model of CpomPBP2^18^. Fluorometric analyses were performed using a Hitachi F-4500 spectrofluorimeter in a 1cm light path fluorimeter quartz cuvette. To measure the intrinsic tryptophan fluorescence, an excitation wavelength of 250 nm was chosen and the emission scans were recorded from 280 to 400 nm with a slit width of 5 nm. Spectra were recorded with 2 μM CpomPBP2 in 50 mM Tris-HCl of different pH value at 25 °C^16^,^20^,^33^.

### Virtual screening based on molecular docking simulations

To obtain the suitable binding poses of CpomPBP2 with the 31 compounds (see Supplementary Table S1) composed of codling moth pheromones and host volatiles reported hitherto^52^,^53^, and to rank their binding affinity, virtual screening based on molecular docking was investigated by the program GOLD 5.3 54. To find out the best set of docking parameters and to ensure reliability of docking results, the bombykol derived from the crystal structure of the PBP-bombykol complex was first docked into the binding site of the CpomPBP2 3D model. The C25 atom coordinates of the bombykol were defined as the centroid of the binding site with 10 Å radius sphere. 3D structures of all compounds were sketched using Maestro version (Schrodinger Inc.) and optimized 2000 steps in Amber12 with the GAFF force field^55^. After being performed 5000 steps minimization in Amber12 with the ff99SB force field^56^, the 3D model of CpomPBP2 was selected as a receptor for docking simulations. For binding pose prediction, ChemPLP was suggested to be superior to other scoring functions in the GOLD program^39^. Hence the ChemPLP score was employed to obtain the most accurate binding modes for the 31 candidate compounds. It should be noted that all docking simulations were done at pH7.0. Interaction diagram was presented by the program Maestro version 10.1 and visualization of the structures was performed by PyMol 1.3r1 edu^57^.

### Competitive binding assay

On the basis of virtual screening and commercial availability, 13 typical ligands were purchased from Aladdin (China), Sigma-Aldrich (USA) and TCI (Japan) to be subjected to competitive binding assay, the major sex pheromone component codlemone (E8, E10-12:OH) was also employed as a control. Fluorescence emission spectra were recorded on a Hitachi F-4500 spectrofluorimeter in a 1 cm light path fluorimeter quartz cuvette. To get the dissociation constant (*K*_*D*_) between 1-NPN and CpomPBP2, 2 μM CpomPBP2 dissolved in 50 mM Tris-HCl pH6.8/pH5.0 was added with 1 mM 1-NPN to final concentrations of 0.5-20 μM. Purchased ligands were then added with increasing concentrations in solutions containing 2 μM CpomPBP2 and 2 μM 1-NPN. The excitation wavelength was decided to be 337 nm and the maximum emission wavelength at 410 nm was recorded. All ligands including 1-NPN were dissolved in GC grade methanol (Aladdin, China).

Ligands screened above were employed to test their affinity to TPBP2 by reference to competitive binding assay. Solutions containing 2 μM TPBP2 were titrated with 1-NPN to final concentrations of 1-32 μM and competitive binding under pH6.8 and pH5.0 was performed following above procedures described.

### Dissociation constants (*K*_*D*_) calculation

It was assumed that CpomPBP2 was 100% active and the binding was 1 : 1 protein : ligand at saturation. In this article, Graphpad Prism software (Graphpad Software, Inc.) was applied to analyze the obtained data. For determination of dissociation constants, the intensity value corresponding to the maximum fluorescence emission was plotted against the concentration of 1-NPN, and bound ligands were evaluated from the values for fluorescence intensity. The curve was linearized using Scatchard Plots to verify the confidence of *K*_*D*_. Dissociation constants of the competitors were calculated from the corresponding IC_50_ value by reference to the following equation (1):


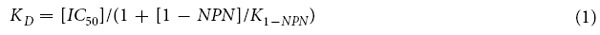


In this equation, [IC_50_] stands for the ligand concentration where the ligand quenching the fluorescence intensity of 1-NPN to 50%, [1-NPN] and *K*_*1-NPN*_ mean the free concentration of 1-NPN and the *K*_*D*_ of the CpomPBP2/1-NPN complex respectively.

### Binding energy calculations

To compare the binding affinity of these semiochemicals to CpomPBP2 3D model, the top docking pose for each analog corresponding to the ChemPLP score was rescored by the Chemscore to measure affinity data by ranking according to the chemscore delta value^58^,^59^. Chemscore estimates the binding free energy ΔG according to equation (2):


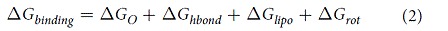


The Chemscore function in our work can be written in the form:


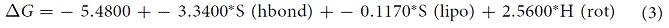


Each component of this equation is the product of a term dependent on the magnitude of a particular physical contribution to free energy (e.g. hydrogen bonding).

### Computational alanine scanning mutagenesis

Based on the competitive binding assay, the complex formed by CpomPBP2 and the ligand with the highest affinity was subjected to the computational alanine scanning (CAS) method^48^ to verify the reliability of the key amino acid residues identified by the simulation docking. The CAS was performed according to our former reports^39^,^60^.

## Additional Information

**How to cite this article**: Tian, Z. *et al.* Structural insights into *Cydia pomonella* pheromone binding protein 2 mediated prediction of potentially active semiochemicals. *Sci. Rep.* **6**, 22336; doi: 10.1038/srep22336 (2016).

## Supplementary Material

Supplementary Information

## Figures and Tables

**Table 1:**
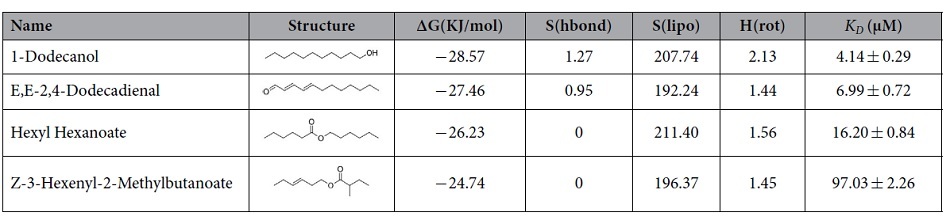
Predicted Gibson free energy change (ΔG), individual energy terms and dissociation constants (*K*_*D*_) for ligands binding to CpomPBP2.

**Table 2:** Oligonucleotide Primers used for cloning and expression of pheromone binding protein 2 (CpomPBP2) from *Cydia pomonella*.

Purpose/Primer Name	Sequence (5′-3′)
Tissue Distribution of CpomPBP2(RT-PCR)	
PBP2F	ATGGCGGCCGCCGCGAAATGG
PBP2R	CTACGACGGCTTGACTTCAGT
ActinF	TCCGGCATGTGCAAGGCCGGT
ActinR	GTCCCAGTTTGTGACGATGCC
CpomPBP2 Expression	
EP2F	CGGGATCCATGTCGGCGGAGATTATGAAAAA
EP2R	CCAAGCTTCTACGACGGCTTGACTTCAGTCA
TPBP2 (C-terminus Truncated CpomPBP2) Expression	
TP2F	CGGGATCCATGTCGGCGGAGATTATG
TP2R	CGAAGCTTCTACGCCCACTTGAGCCC
