# Supplementary material for: Structural insights into Cydia pomonella pheromone binding protein 2 mediated prediction of potentially active semiochemicals
Source: Sci Rep. 2016 Mar 1;6:22336. doi: 10.1038/srep22336 (PMC4772377; doi:10.1038/srep22336)
Supplement: Supplementary Information [file srep22336-s1.pdf]

Structural insights into *Cydia pomonella* pheromone binding protein 2 mediated prediction of potentially active semiochemicals

Zhen Tian[1a], Jiyuan Liu[1a], Yalin Zhang[1]*

[1] Key Laboratory of Plant Protection Resources & Pest Management of the Ministry of Education, College of Plant Protection, Northwest A&F University, Yangling 712100, Shaanxi, China

[a]These authors contributed equally to this work.

*Corresponding author, e-mail: yalinzh@nwsuaf.edu.cn

Ya-Lin Zhang, E-mail: yalinzh@nwsuaf.edu.cn.

*Corresponding Author

Key Laboratory of Plant Protection Resources & Pest Management of the Ministry of Education, Northwest A&F University, Yangling 712100, Shaanxi, China.

Tel./Fax: +86-29-8709-2190.

E-mail: yalinzh@nwsuaf.edu.cn.

Supplementary Information

Supplementary Fig. S1

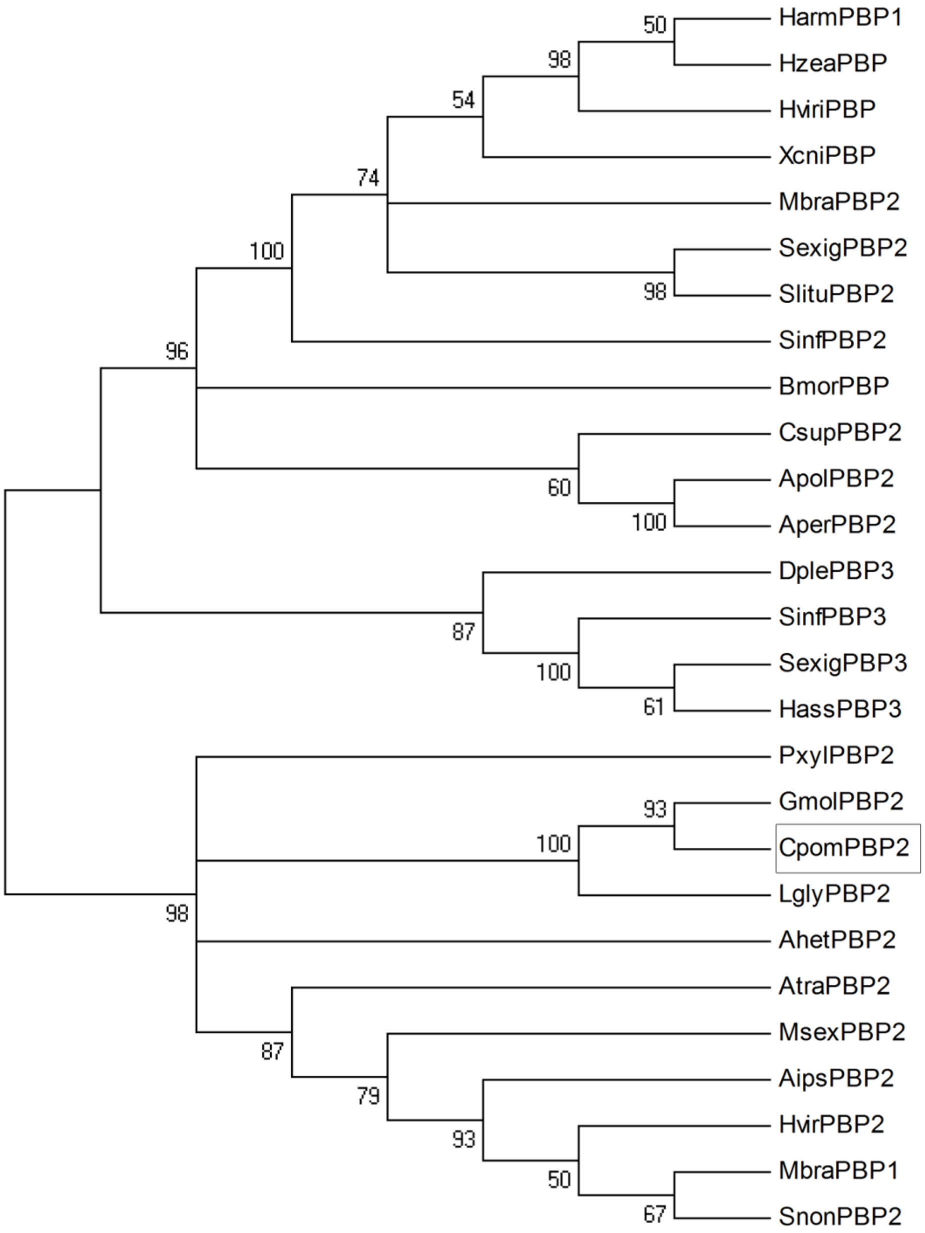

Supplementary Fig. S2

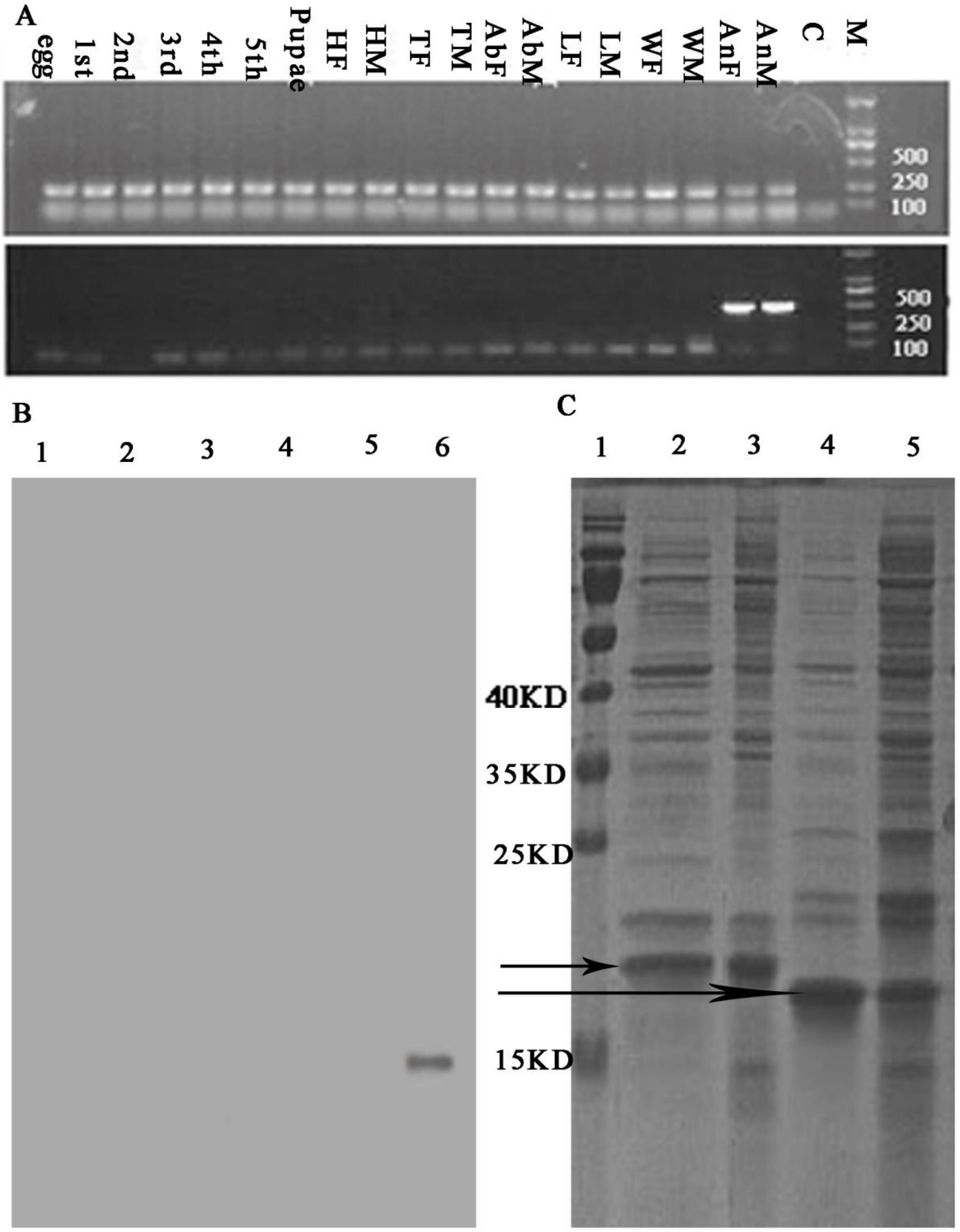

Supplementary Fig. S3

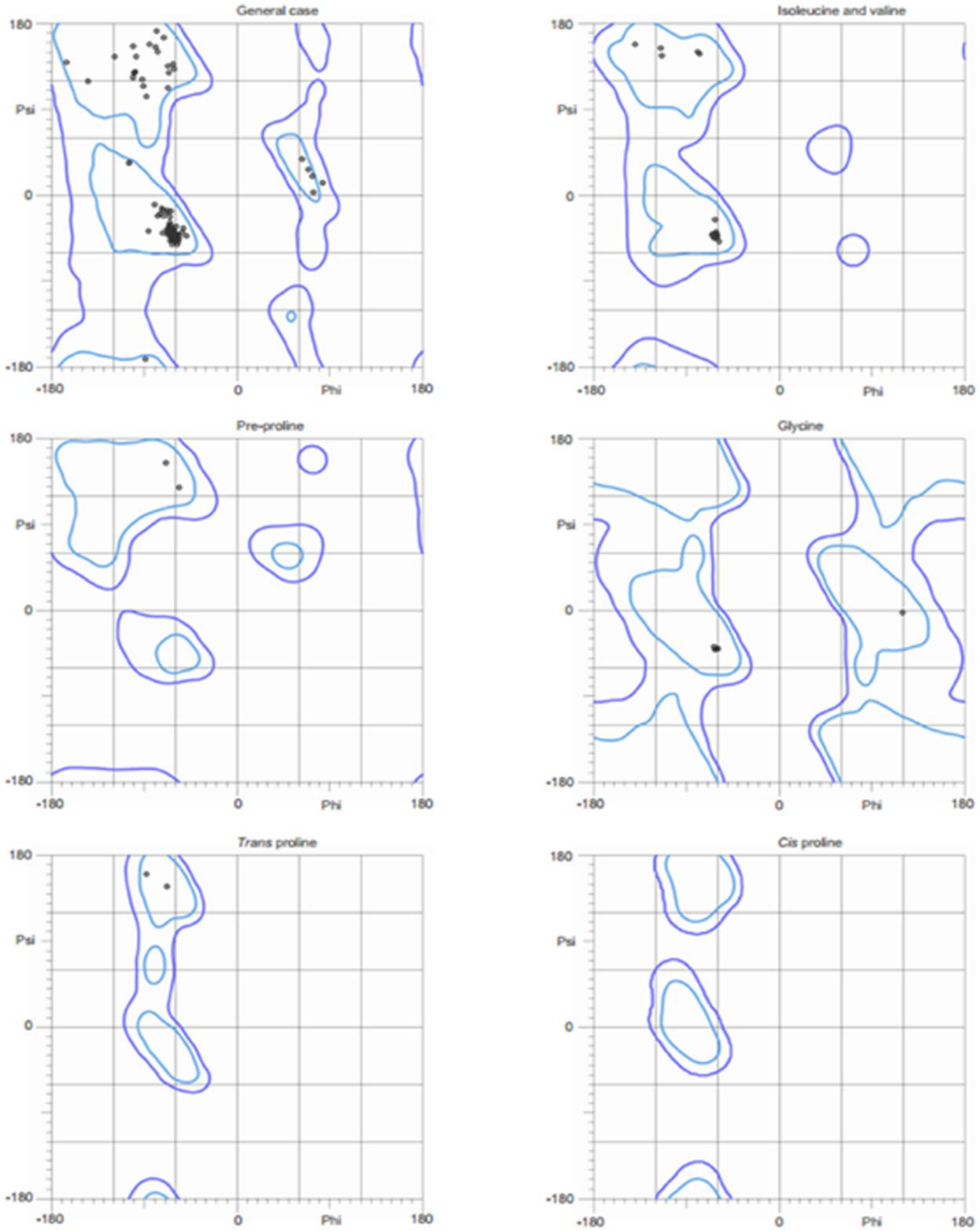

Supplementary Fig. S4

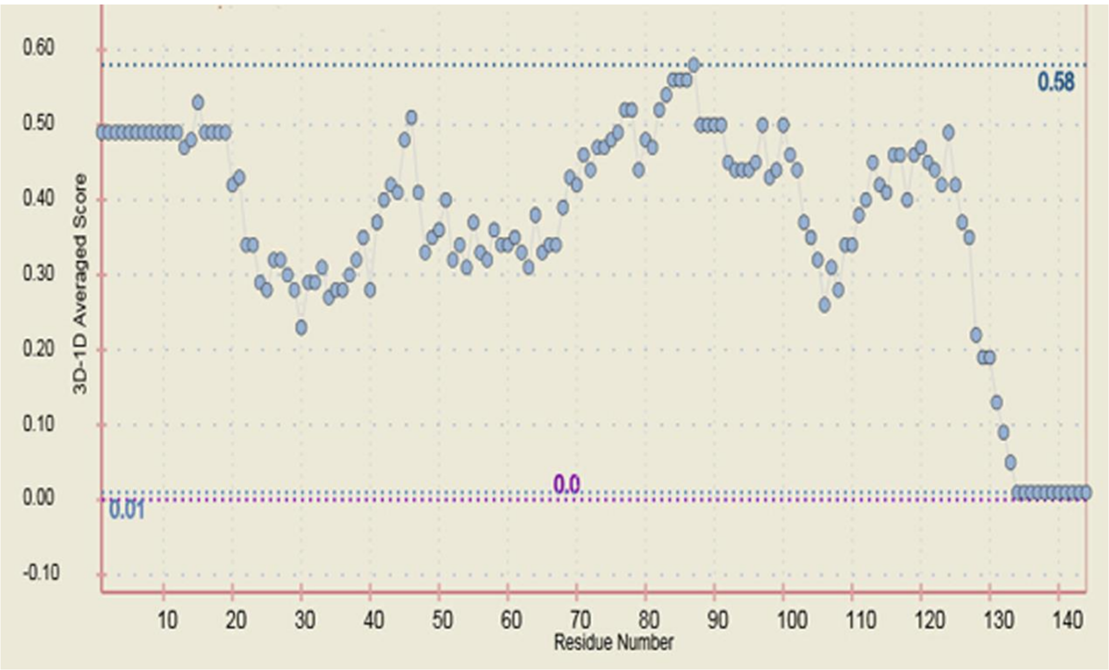

Supplementary Fig. S5

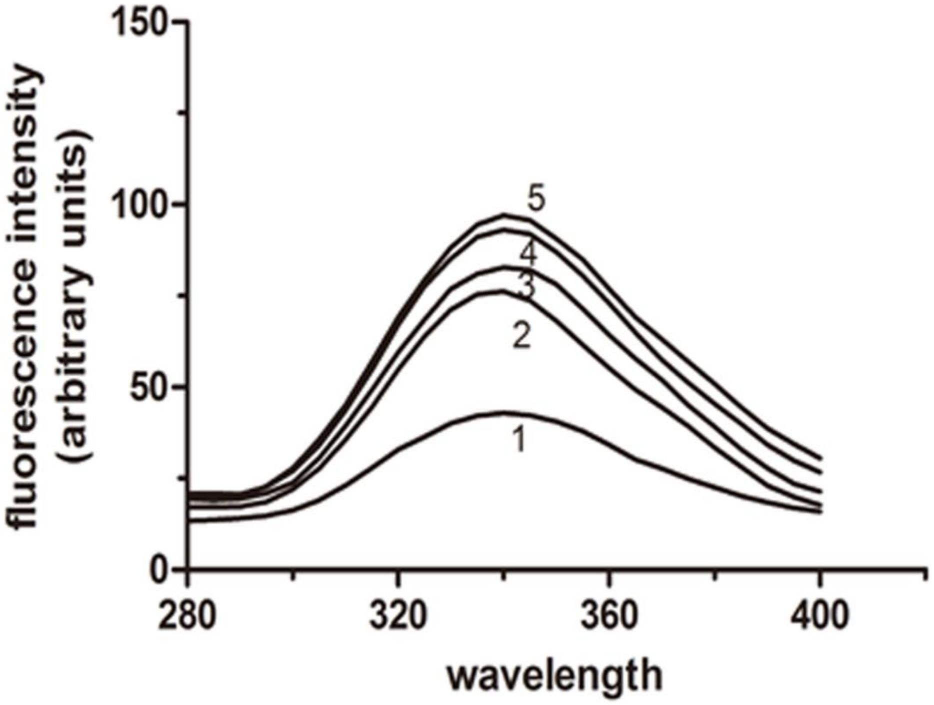

Supplementary Fig. S6

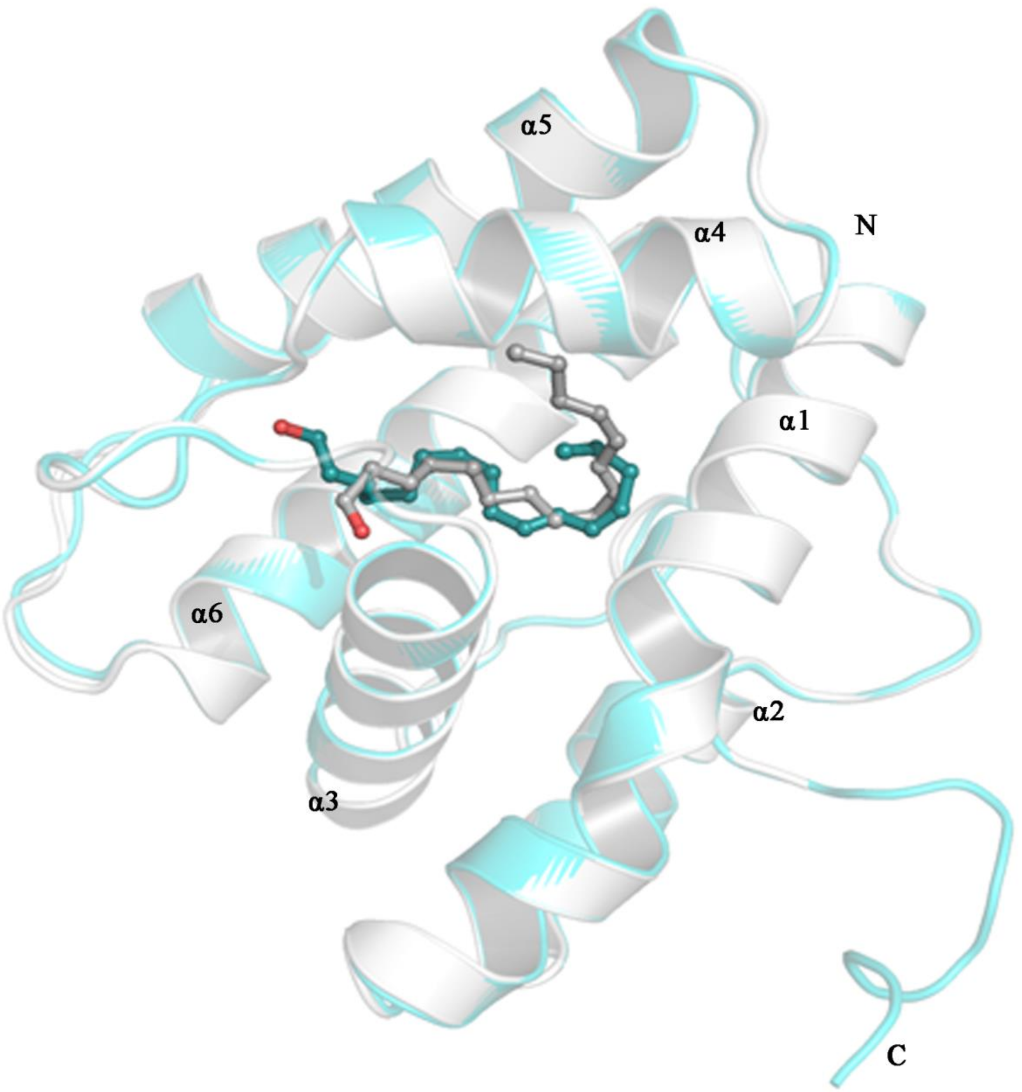

Supplementary Fig. S7

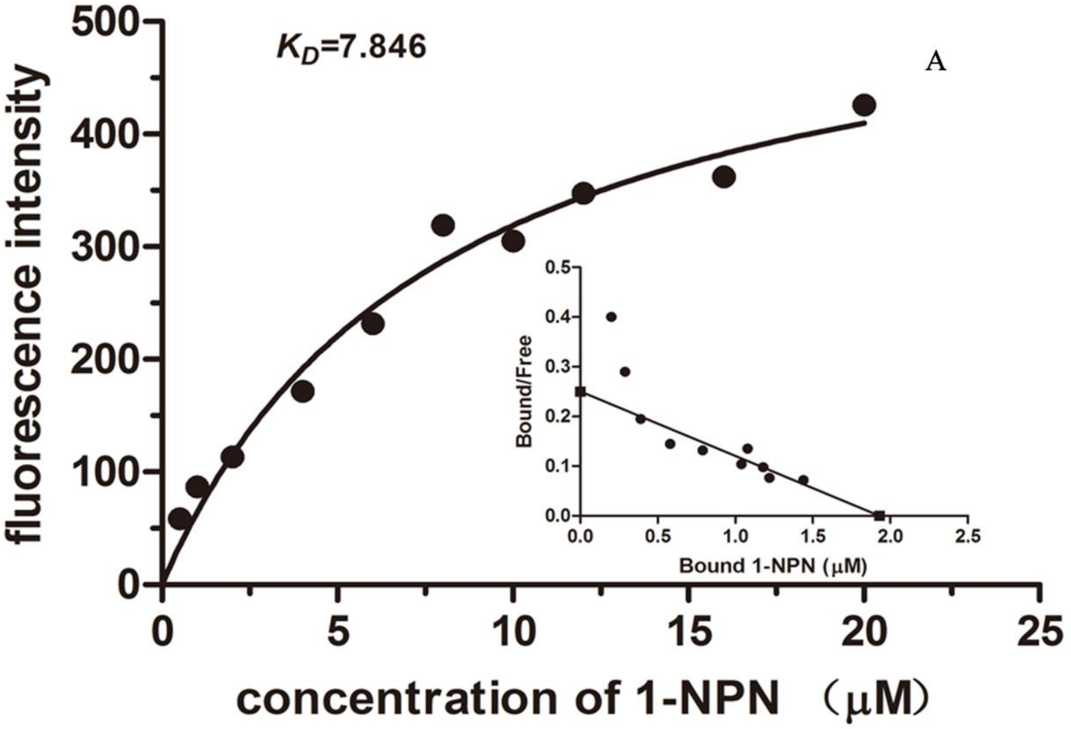

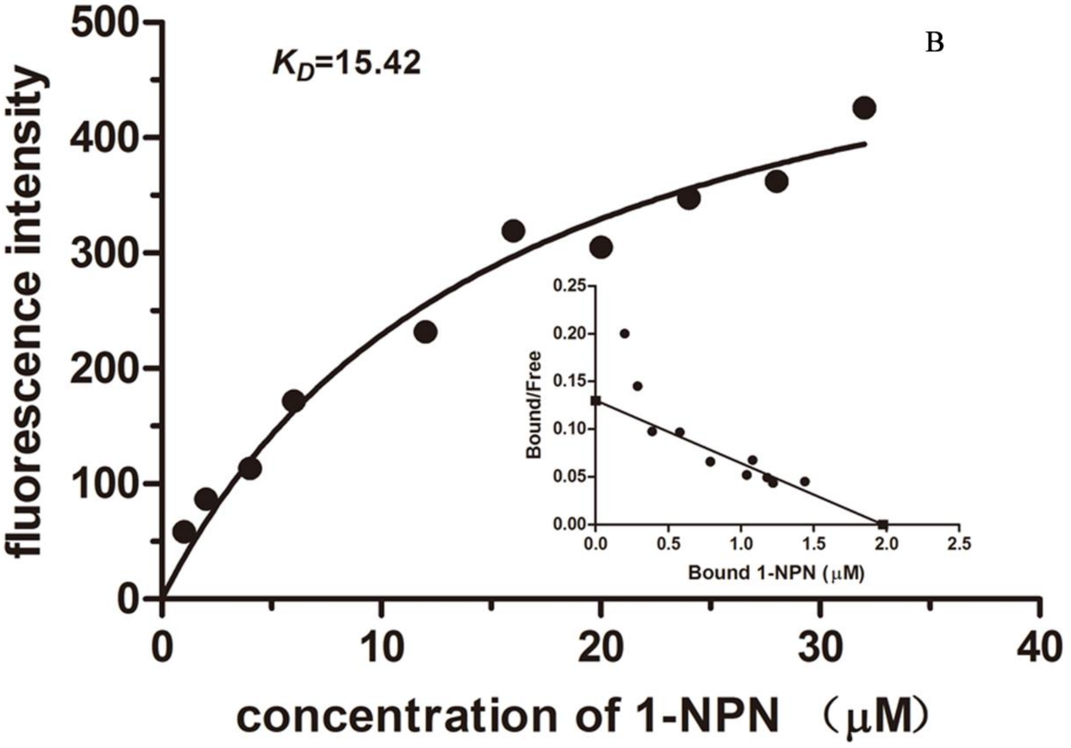

Supplementary Fig. S8

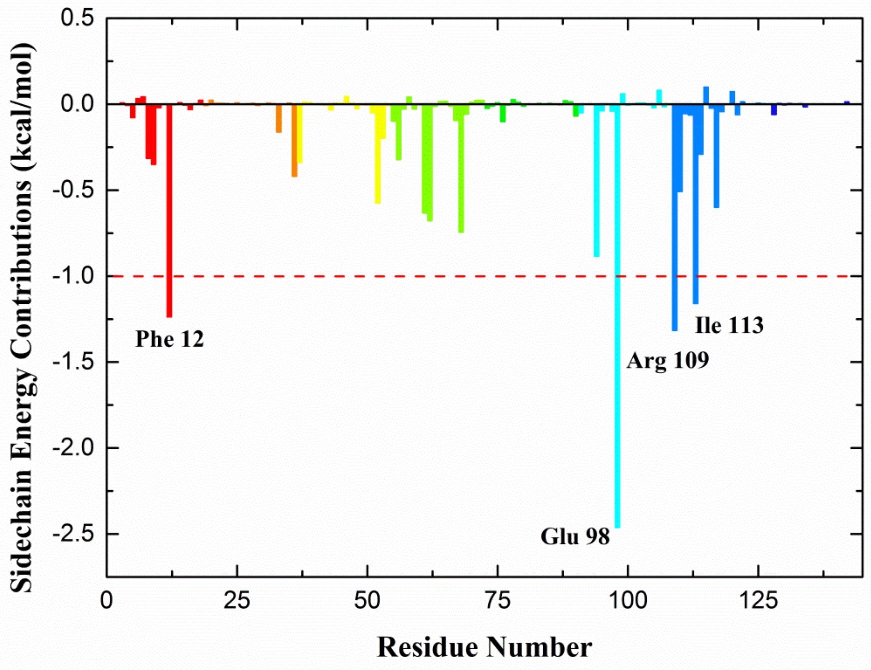

Supplementary Figure legends

Supplementary Fig. S1 Phylogenetic relationship of CpomPBP2 with the representative sequences from previously described lepidopteran PBPs. The tree was constructed with the neighbor-joining method and CpomPBP2 was marked with box. Bootstrap analysis used 1000 replicates.

Supplementary Fig. S2 Expression of CpomPBP2. (A) RT-PCR analysis of β-actin and CpomPBP2expression. cDNAs templates from egg, the 1st to 5th instar larva (1st to 5th), pupae, female and male head (HF/HM), female and male thorax (TF/TM), female and male abdomen (AbF/AbM), female and male leg (LF/LM), female and male wing (WF/WM), female and male antennae (AnF/AnM). (B) Western blot analysis of CpomPBP2 from different tissues. Lanes 1 to 6 were head, thorax, abdomen, leg, wing and antennae. (C) Prokaryotic expression of CpomPBP2 and TPBP2. Lanes 1 to 5 were Marker, supernatant and inclusion body of CpomPBP2, and supernatant and inclusion body of TPBP2.

Supplementary Fig. S3 Ramachandran plot of the CpomPBP2 model. The plot showed that 99.3% (141/142) of all residues were in favored (98%) regions, 100.0% (142/142) of all residues were in allowed (>99.8%) regions, and there were no outliers.

Supplementary Fig. S4 Profile 3D score of the CpomPBP2 model. The result showed that 88.89% of the residues had an averaged 3D-1D score >= 0.2, and at least 80% of the amino acids have scored >= 0.2 in the 3D/1D profile.

Supplementary Fig. S5 Intrinsic fluorescence analyses of CpomPBP2 under different pH value. Spectra of 2μM CpomPBP2 were taken in 50mM Tris-HCl, pH 4.5 (Spectrum 1), pH 5.5 (Spectrum 2), pH 6.5 (Spectrum 3), pH7.5 (Spectrum 4), pH8.5 (Spectrum 5).

Supplementary Fig. S6 The superimposition of the conformations between CpomPBP2-bombykol and BmorPBP-bombykol complexes. The superimposition RMSD with the value of 0.113Å between the conformations of the bombykol in BmorPBP and CpomPBP2

indicated the accurate docking of the whole compounds in the binding pocket of CpomPBP2.

Supplementary Fig. S7 Binding of N-phenyl-1-naphthylamine (1-NPN) to CpomPBP2 (A) and TPBP2 (B). Protein was 2μM in 50mM Tris-HCl, pH 6.8. Aliquots of a 1mM 1-NPN in methanol were added to the protein to final concentrations of 0.5-20μM for CpomPBP2 and 1-32μM for TPBP2. Solutions were excited at a wavelength of 337nm and the maximum emission spectra were recorded at ~410nm. The binding curves and the relative Scatchard plots indicated a binding constant of 7.846μM for CpomPBP2 and 15.42μM for TPBP2.

Supplementary Fig. S8 Residue-ligand interaction spectrum of 1-Dodecanol/CpomPBP2 complex according to the MM-GBSA method. The x-axis denotes the residue number of the CpomPBP2 and the y-axis denotes the sidechain energy contribution for each residue.

Supplementary Table S1 The ChemPLP fitness and predicted individual energy terms for the ligands subjected to virtual screening.

Name	index	CAS NO.	Structure	ΔG(KJ/mol)	S(hbond)	S(lipo)	H(rot)	PLP Fitness
Bombykol	1	765-17-3	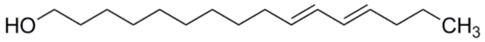	-36.6153	0.9991	284.8378	2.1592	76.6429
*1-Dodecanol	2	27342-88-7	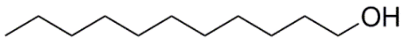	-28.5689	1.2688	207.7409	2.1308	59.2
*E,E-2,4-Dodecadienal	3	21662-16-8	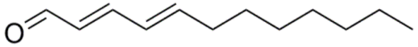	-27.464	0.9527	192.239	1.4414	55.6848
Z-3-Hexenyl Hexanoate	4	31501-11-8	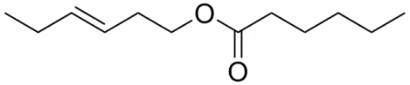	-26.8011	0	215.6226	1.5261	59.0227
E,Z-2,6-Dodecadienal	5	21662-13-5	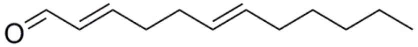	-26.4807	0.9987	187.225	1.6564	57.9448
*Hexyl Hexanoate	6	6378-65-0	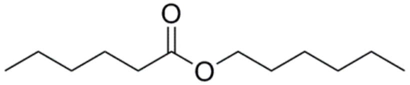	-26.2264	0	211.4047	1.5578	57.4725
Ethyl 2,4-Decadienoate	7	3025-30-7	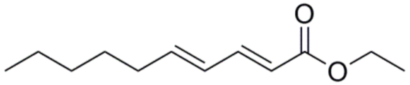	-25.6823	0	202.3782	1.3578	55.1715
*Z-3-Hexenyl-2-Methylbutanoate	8	53398-85-9	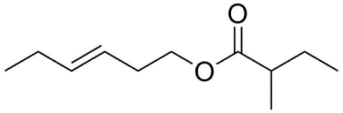	-24.7418	0	196.371	1.4506	55.0892
Hexyl Butyrate	9	2639-63-6	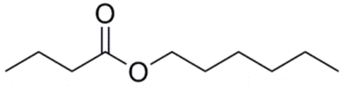	-24.5498	0.9988	168.8557	1.5712	49.8747

Hexyl- 2-Methylbutyrate	10	10032-15-2	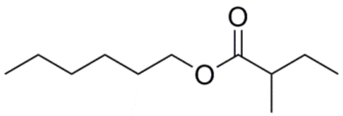	-24.4549	0	194.9309	1.4969	53.4008
Butyl Octanoate	11	589-75-3	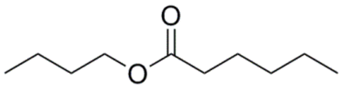	-23.9003	0	191.9646	1.578	55.7864
Methyl-2-Butylhexanoate	12	2601-13-0	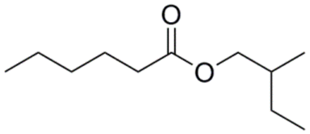	-23.6884	0	187.9682	1.4781	53.5109
3-Methylbutyl Hexanoate	13	2198-61-0	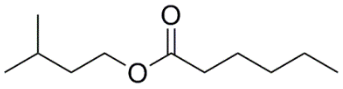	-22.9692	0	181.9439	1.4837	51.3727
*Isopentyl Hexanoate	14	540-07-8	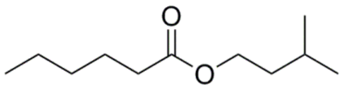	-22.9038	0	182.5947	1.539	52.7154
Butyl Hexanoate	15	626-82-4	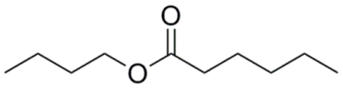	-22.6035	0	179.7088	1.5244	49.909
E-3-Hexenyl Butyrate	16	53398-84-8	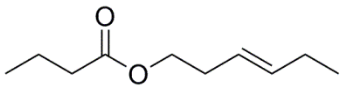	-21.8328	0	172.9871	1.5183	50.2438
1-Hexyl Propionate	17	2445-76-3	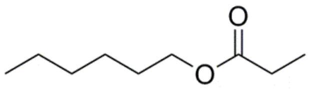	-21.5305	1	143.8431	1.609	46.7335

*Isobutyl Hexanoate	18	105-79-3	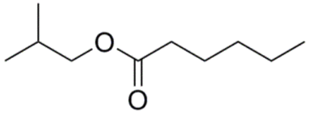	-20.8548	0	163.4025	1.4622	48.0159
*E-2-Hexenyl Acetate	19	2497-18-9	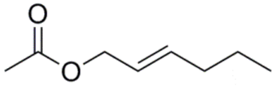	-20.5757	0.9992	135.5804	1.6033	43.3959
2-Methylbutyl Butyrate	20	51115-64-1	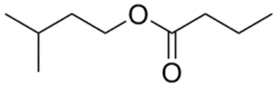	-19.9939	0	155.7257	1.4477	45.947
*Z-3-Hexenyl Acetate	21	3681-71-8	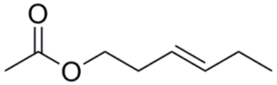	-19.8262	0.9996	129.3057	1.6099	44.1452
*Butyl Valerate	22	591-68-4	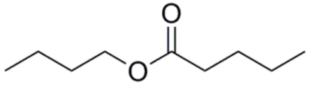	-19.2563	0	150.582	1.5007	45.7204
*Pentyl Butyrate	23	540-18-1	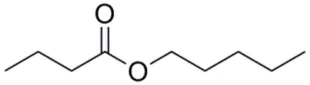	-19.1818	0	150.545	1.5281	46.7245
*E-3-Hexenol	24	928-97-2	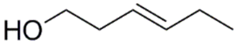	-18.7824	0.974	122.6804	1.6812	36.7676
Z-2-Hexenol	25	928-94-9	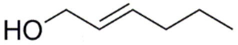	-18.2548	0.9843	117.6454	1.6708	36.7851
2-Methylbutyl Propionate	26	2438-20-2	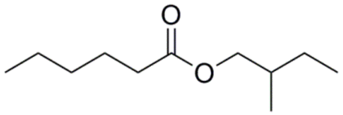	-18.103	0	139.6381	1.451	42.1665

Butyl Propionate	27	590-01-2	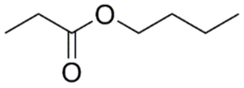	-18.0947	0.998	111.7072	1.4798	38.4971
*Butyl Butyrate	28	539-90-2	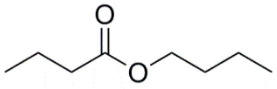	-17.7965	0	135.9172	1.4007	43.219
Butyl Butanoate	29	109-21-7	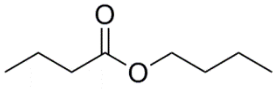	-17.6397	0	136.3815	1.4832	42.2514
*Ethyl-2-Methylbutyrate	30	7452-79-1	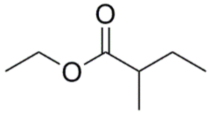	-15.4751	0	114.9667	1.35	36.4293
Hexyl Formate	31	629-33-4	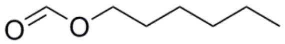	-14.8159	0.995	105.8991	2.4913	40.6362

Supplementary Table S2 The theoretical $\Delta\Delta G_{bind}$[a] value for WT and mutant complexes.

Protein	F12A	E98A	R109A	I113A
1-Dodecanol-CpomPBP2				
$\Delta\Delta G_{bind}$	3.17(0.63)	5.34(1.45)	6.25(1.94)	2.66(0.53)

[a] All values are given in kcal/mol with standard errors in parentheses.
